# Use of Ambroxol as Therapy for Gaucher Disease

**DOI:** 10.1001/jamanetworkopen.2023.19364

**Published:** 2023-06-21

**Authors:** Xia Zhan, Huiwen Zhang, Gustavo H. B. Maegawa, Yu Wang, Xiaolan Gao, Dengbin Wang, Jinning Li

**Affiliations:** 1 Department of Pediatric Endocrinology and Genetics, Xinhua Hospital, Shanghai Institute for Pediatric Research, Shanghai Jiao Tong University School of Medicine, Shanghai, China; 2 Vagelos College of Physicians and Surgeons, Columbia University, Department of Pediatrics, Columbia University Medical Center, New York, New York; 3 Department of Radiology, Xinhua Hospital, Shanghai Jiao Tong University School of Medicine, Shanghai, China

## Abstract

**Importance:**

Ambroxol was identified as an enhancer of stability and residual activity of several misfolded glucocerebrosidase variants in 2009.

**Objectives:**

To assess hematologic and visceral outcomes, biomarker changes, and safety of ambroxol therapy for patients with Gaucher disease (GD) without disease-specific treatment.

**Design, Setting, and Participants:**

Patients with GD who could not afford enzyme replacement therapy were enrolled and received oral ambroxol from May 6, 2015, to November 9, 2022, at Xinhua Hospital, affiliated with Shanghai Jiao Tong University School of Medicine, Shanghai, China. Thirty-two patients with GD (29 with GD type 1, 2 with GD type 3, and 1 with GD intermediate types 2-3) were enrolled. Of those, 28 patients were followed up for longer than 6 months; 4 were excluded due to loss of follow-up. Data analyses were performed from May 2015 to November 2022.

**Intervention:**

An escalating dose of oral ambroxol (mean [SD] dose, 12.7 [3.9] mg/kg/d).

**Main Outcomes and Measures:**

Patients with GD receiving ambroxol were followed up in a genetic metabolism center. Biomarkers of chitotriosidase activity and glucosylsphingosine level, liver and spleen volumes, and hematologic parameters were measured at baseline and various time points throughout the ambroxol treatment.

**Results:**

A total of 28 patients (mean [SD] age, 16.9 [15.3] years; 15 male patients [53.6%]) received ambroxol for a mean (SD) duration of 2.6 (1.7) years. Two patients with severe symptoms at baseline experienced deterioration of hematologic parameters and biomarkers and were deemed nonresponders; clinical response was observed in the other 26 patients. After 2.6 years of ambroxol treatment, the mean (SD) hemoglobin concentration improved from 10.4 (1.7) to 11.9 (1.7) g/dL (mean [SD], 1.6 [1.7] g/dL; 95% CI, 0.8-2.3 g/dL; *P* < .001), and the mean (SD) platelet count improved from 69 (25) to 78 (30) × 10^3^/µL (mean [SD], 9 [22] × 10^3^/µL; 95% CI, −2 to 19 × 10^3^/µL; *P* = .09). The mean (SD) spleen volume decreased from 17.47 (7.18) to 12.31 (4.71) multiples of normal (MN) (mean [SD], −5.16 [5.44] MN; 95% CI, −10.19 to −0.13; *P* = .04), and the mean (SD) liver volume decreased from 1.90 (0.44) to 1.50 (0.53) MN (mean [SD], −0.39 [0.42] MN; 95% CI, −0.75 to −0.04; *P* = .03). Biomarker median percentage changes from baseline were −43.1% for chitotriosidase activity (from 14 598 [range, 3849-29 628] to 8312 [range, 1831-16 842] nmol/mL/h; *z* = −3.413; *P* = .001) and −34.1% for glucosylsphingosine level (from 251.3 [range, 73.6-944.2] to 165.7 [range, 21.3-764.8] ng/mL; *z* = −2.756; *P* = .006). Patients were divided into subgroups according to age when initiating treatment; those who received treatment at a younger age (mean [SD] age, 6.3 [2.7] years) experienced more rapid improvements: hemoglobin concentration increased by 16.5% (from 10.3 [1.5] to 12.0 [1.5] g/dL; mean [SD] change, 1.6 [1.6] g/dL; 95% CI, 0.7-2.5 g/dL; *P* = .002), and platelet count increased by 12.0% (from 75 [24] to 84 [33] × 10^3^/µL; mean [SD] change, 9 [26] × 10^3^/µL; 95% CI, −5 to 24 × 10^3^/µL; *P* = .17); whereas chitotriosidase activity decreased by 64.0% (from 15 710 [range, 4092-28 422] to 5658 [range, 1146-16 843] nmol/mL/h; *z* = −2.803; *P* = .005), and glucosylsphingosine level decreased by 47.3% (from 248.5 [range, 122.8-674.9] to 131.0 [range, 41.1-448.5] ng/mL; *z* = −2.385; *P* = .02). Three of the 28 patients experienced mild and transient adverse events.

**Conclusions and Relevance:**

In this case series of ambroxol repurposing among patients with GD, long-term treatment with ambroxol was safe and associated with patient improvement. Improvements in hematologic parameters, visceral volumes, and plasma biomarkers were larger among patients with relatively mild symptoms of GD and patients who received initial treatment at younger ages.

## Key Points

**Question:**

What are the biochemical changes associated with ambroxol repurposing among patients with Gaucher disease who have not received any disease-specific treatment?

**Findings:**

In this case series of 28 unselected participants, significant improvements in hematologic parameters, visceral volumes, and plasma biomarkers were observed for 26 patients after a mean (SD) treatment duration of 2.6 (1.7) years.

**Meaning:**

Long-term treatment with ambroxol was associated with patient improvement; improvements in hematologic parameters, visceral volumes, and plasma biomarkers were larger among patients with relatively mild symptoms of Gaucher disease and patients who received initial treatment at younger ages.

## Teaser

This case series assesses hematologic and visceral outcomes, biomarker changes, and safety of ambroxol therapy for patients with Gaucher disease without disease-specific treatment.

## Introduction

Gaucher disease (GD) is one of the most common lysosomal storage disorders caused by deficiency in the enzyme β-glucocerebrosidase because of variants in the *GBA1* (OMIM 606463) gene, resulting in glucosylceramide accumulation. Three main clinical types of GD are generally characterized based on the absence or presence of central nervous system involvement: nonneuronopathic type 1 (GD1), acute neuronopathic type 2 (GD2), and chronic neuronopathic type 3 (GD3). The incidence of GD in the general population is 1 in 50 000 to 100 000.^1^ The pilot newborn screening for GD in Shanghai reported that the incidence in east China is approximately 1 in 80 855.^2^

Two therapeutic approaches have been approved for treating GD: enzyme replacement therapy (ERT) and substrate reduction therapy (SRT).^3^ Visceral and hematologic abnormalities were significantly improved with ERT.^4,5^ Unfortunately, the effectiveness of ERT for neurologic symptoms of GD is almost negligible due to the inability of the enzyme to cross the blood-brain barrier (BBB).^6,7^ Miglustat and eliglustat could cross the BBB but failed to show significant benefits for neurologic manifestations.^8,9^ Health insurance for ERT is not fully covered in the People’s Republic of China, and some patients for whom ERT is indicated must pay for it themselves. Some patients cannot receive efficacious treatment due to the prohibitive costs of ERT or SRT.

Unlike the current drugs used to treat GD, which are very expensive and do not cross the BBB, new small-molecule therapies that enhance glucocerebrosidase are being developed to overcome these limitations.^10^ Ambroxol is a widely used cough suppressant and mucolytic with an excellent safety profile.^11,12^ In 2009, ambroxol was identified in a high-throughput screening of a regulatory-approved compound collection as an enhancer of stability and residual activity of several misfolded glucocerebrosidase variants.^13^ Later, a pilot open-label study with 12 adult patients with GD1 naive to any disease-specific treatment showed some improvements in disease parameters after the patients received ambroxol for 6 months.^14^

Ambroxol has been shown to cross the BBB.^11,15^ Based on this evidence, in several countries where ambroxol is approved and available, it has been prescribed off label with ERT or SRT for patients with neuronopathic GD (nGD).^11,16,17^ With no specific treatment for nGD and the aforementioned encouraging reports, off-label use of high-dose ambroxol has become a realistic option for managing nGD as an adjunct to ERT or for GD among patients without access to ERT or SRT. This study investigated the observational data of ambroxol repurposing in a large case series of patients without GD-specific treatment and its association with hematologic parameters, visceral volumes, and biomarkers of GD.

## Methods

### Patients

The local institutional research ethics committees approved the study at Xinhua Hospital, affiliated with Shanghai Jiao Tong University School of Medicine, Shanghai, China. Written informed consent was obtained from all participants aged 18 years or older and from parents of patients younger than 18 years. This study followed the Appropriate Use and Reporting of Uncontrolled Case Series in the Medical Literature reporting guideline for case series studies.

Patients with GD who could not afford ERT due to the prohibitive cost were enrolled and received oral ambroxol from May 6, 2015, to November 9, 2022. All patients were confirmed to have GD by measuring leukocyte lysosomal acid β-glucosidase activity within the affected range and *GBA1* biallelic pathogenic variants. Patients were recommended to be followed up once every 6 months. Patients were assessed at baseline and scheduled follow-up visits, including disease status inquiry, physical examination, recording adverse events, routine blood tests, and measuring chitotriosidase activity and glucosylsphingosine level. Spleen and liver volumes were derived from volumetric magnetic resonance imaging if available and measured in multiples of normal (MN).

Ambroxol administration was initiated with a starting dose of 5 mg/kg/d divided into 3 equal doses. It was suggested to increase the doses gradually according to clinical outcomes of ambroxol at follow-up visits, no more than the maximal dose (25 mg/kg). Patients received an escalating dose of oral ambroxol (mean [SD] dose, 12.7 [3.9] mg/kg/d) at the last visits.

We evaluated the achievement of the following therapeutic goals: spleen volume, 8 MN or less or a 50% or more decrease from baseline; liver volume, 1.5 MN or less or a 30% or more decrease from baseline; hemoglobin, 11.0 g/dL or more for women and 12.0 g/dL or more for men (to convert to grams per liter, multiply by 10.0); and platelet count, 100 × 10^3^/µL or more or double the baseline value for patients with a platelet count less than 60 × 10^3^/µL (to convert to ×10^9^ per liter, multiply by 1.0) at baseline.^18^

### Chitotriosidase Activity

Chitotriosidase activity was measured in plasma samples by a spectrofluorometric method using the synthetic substrate 4-methylumbelliferyl 4-deoxy-β-D-chitobiose (Carbosynth Limited). Fluorometric measurements were performed at excitation λ = 365 nm and emission λ = 460 nm (PerkinElmer). Plasma samples were stored at −80 °C until analysis.

### Glucosylsphingosine Assay

Dried blood spot glucosylsphingosine level was measured for 1 patient, while plasma glucosylsphingosine level was monitored throughout treatment for the other patients. Glucosylsphingosine was measured on ultra-performance liquid chromatography using a Xevo TQ S detector (Waters). The glucosylsphingosine standard and the internal standard that was used were purchased from Matreya Inc. Plasma samples were collected in heparin tubes and frozen at −80 °C. In brief, in a 1.5-mL vial, 30 μL of plasma sample was mixed with 200 μL of internal standard solution. Samples were vortexed and centrifuged (12 000*g* for 5 minutes), and then the supernatant was transferred to a 96-well plate.

Dried blood spot samples were collected on filter cards and frozen at −80 °C. One 5-mm blood spot and each dried blood spot calibrator, control, and sample were punched into a 96-well plate. Then 200 μL of the internal standard working solution was added to each vial. The plate was sealed with aluminum foil tape and shaken for 30 minutes at room temperature in an incubator-shaker unit. The supernatant was transferred to a 96-well plate.

Analytes were separated on an Acquity BEH Amide column (column dimensions, 2.1 × 100 mm; particle size, 1.7 μm; Waters). Mass spectrometry was performed in the positive ionization mode using an electrospray ionization source, with the following transitions: the mass number of an ion divided by its charge number (ie, the mass to charge ratio) is 462.3 > 264.3 for glucosylsphingosine and 468.3 > 264.3 for glucosylsphingosine-C6.

### Statistical Analysis

Data was collected from May 2015 to November 2022. For statistical calculations and data collection, SPSS, version 17 (SPSS Inc) was used. Data distribution was assessed. A 2-sided Wilcoxon signed rank test was used for chitotriosidase activity and glucosylsphingosine level at baseline and follow-up visits; a paired *t* test was used for hematologic and visceral parameters. A 2-sided *P* < .05 was considered statistically significant. Graphs were made in GraphPad Prism, version 5 (GraphPad Software).

## Results

### Study Cohort

Thirty-two patients with GD, including 29 with GD1, 2 with GD3, and 1 with GD intermediate type 2-3, were enrolled and treated with ambroxol. Patients with a follow-up duration of fewer than 6 months were excluded from the analysis. Four of 32 patients with GD1 (cases 29-32) were lost to follow-up and were excluded from analyses (eFigure 1 in Supplement 1). A total of 28 patients (mean [SD] age, 16.9 [15.3] years; 15 male patients [53.6%]) received ambroxol for a mean (SD) duration of 2.6 (1.7) years (Table 1). Five patients were splenectomized. At baseline, glucosylsphingosine levels were markedly increased in all patients, with a median elevation of up to 240-fold (range, 67-fold to 614-fold). Eleven patients had chitotriosidase values less than 47 nmol/mL/h at baseline, likely reflecting no chitotriosidase enzyme activity due to homozygosity of the common variant 24-bp duplication in the *CHIT1* (OMIM 600031) gene. Baseline chitotriosidase activity was elevated in the other 17 patients, with a median elevation of up to 290-fold (range, 82-fold to 630-fold). Genotypes of patients are summarized in Table 2. The eTable in Supplement 1 presents all patients’ demographic data, clinical features at baseline, ambroxol doses, therapy lengths, adverse events, and clinical improvements. Three of the 28 patients experienced mild and transient adverse events, including nausea, salivation, diarrhea, and rash. Clinical improvements, including reduced fatigue, more energy, fewer nosebleeds, disappearance of petechia on skin as described by the patients or parents, and stable or improved neurologic status as assessed by the physicians during a physical examination, were recorded for 26 of 28 patients (eTable in Supplement 1). Two patients with more severe symptoms (case 11: spleen volume, 41 MN; case 28: spleen volume, 87 MN) experienced deterioration of hematologic parameters and biomarkers and showed no improvement in clinical presentation, and treatment was considered nonefficacious in the study. These 2 patients were not included in the hematologic and biomarker analysis.

**Table 1.** Baseline Characteristics of Patients

Characteristic	Enrolled patients		Patients included in final analysis			
	Total enrolled patients (N = 32)	Patients (duration >6 mo, n = 28)^a^	Patients included in final analysis (n = 26)	Splenectomized patients (n = 5)	Patients by age when starting ambroxol^b^	
					<18 y (n = 15)	≥18 y (n = 6)
Sex, No. (%)						
Male	15 (46.9)	15 (53.6)	14 (53.8)	3 (60.0)	8 (53.3)	2 (33.3)
Female	17 (53.1)	13 (46.4)	12 (46.2)	2 (40.0)	7 (46.7)	4 (66.7)
Age at start of ambroxol, mean (SD), y	17.2 (16.5)	16.9 (15.3)	18.1 (16.6)	37.2 (11.9)	6.3 (2.7)	31.3 (8.3)
Spleen status, No. (%)						
Intact	27 (84.4)	23 (82.1)	21 (80.8)	0	15 (100)	6 (100)
Underwent splenectomy	5 (15.6)	5 (17.9)	5 (19.2)	5 (100)	0	0
Children (<18 y), No./total No. (%)	20/32 (62.5)	17/28 (60.7)	15/26 (57.7)	0	15/15 (100)	0
Hemoglobin, mean (SD), g/dL	10.6 (1.8)	10.7 (1.8)	10.4 (1.7)	12.7 (1.3)	10.3 (1.5)	10.6 (2.4)
Platelet count, mean (SD), ×10^3^/µL	99 (89)	102 (92)	104 (96)	243 (156)	75 (24)	56 (24)
White blood count, mean (SD), /μL	5870 (3170)	6190 (3250)	6130 (3340)	11 600 (2400)	5810 (1680)	2840 (1050)
Chitotriosidase, median (range), nmol/mL/h^c^	13 634 (3849-29 628)^d^	13 634 (3849-29 628)^e^	14 598 (3849-29 628)^f^	22 568 (15 507-29 628)^g^	15 710 (4092-28 422)^h^	10 276 (3849-17 607)^i^
Glucosylsphingosine, median (range), ng/mL^j^	282.8 (73.6-944.2)	265.1 (73.6-944.2)	251.3 (73.6-944.2)	335.5 (73.6-944.2)	248.5 (122.8-674.9)	271.3 (179.8-549.7)

SI conversion factors: To convert hemoglobin to grams per liter, multiply by 10.0; platelet count to ×10^9^ per liter, multiply by 1.0; and white blood cell count to ×10^9^ per liter, multiply by 0.001.

^a^ Excludes 4 patients lost to follow-up.

^b^ Excludes 5 splenectomized patients.

^c^ Normal range, 3 to 47 nmol/mL/h.

^d^ Twelve patients with no chitotriosidase activity were excluded.

^e^ Eleven patients with no chitotriosidase activity were excluded.

^f^ Ten patients with no chitotriosidase activity were excluded.

^g^ Two patients with no chitotriosidase activity were excluded.

^h^ Five patients with no chitotriosidase activity were excluded.

^i^ Two patients with no chitotriosidase activity were excluded.

^j^ Normal ranges, less than 1.1 ng/mL in plasma sample, less than 10 ng/mL in dried blood spot sample.

**Table 2.** Summary of Genotypes Among Patients

Patient No./sex	Type	GBA variant
1/F	GD1	c.1066 delC(L356Wfs*8)/c.1174C>T(R392W)
2/M	GD1	c.226T>G(F76V)/c.1448T>C(L483P)
3/M	GD1	c.226T>G(F76V)/c.1448T>C(L483P)
4/M	GD1	c.680A>G(N227S)/c.1448T>C(L483P)
5/M	GD1	c.926G>A(W309X)/c.223A>G(M75V)
6/F	GD1	c.475C>T(R159W)/c.475C>T(R159W)
7/M	GD1	c.562C>T(L188F)/c.1448T>C(L483P)
8/M	GD1	c.1331A>T(D444V)/E3-10 deletion
9/M	GD1	c.907C>A(L303I)/c.971G>A(R324H)
10/F	GD1	c.587A>G(K196R)/c.680A>G(N227S*)
11/F	GD1	c.260G>A(R87Q)/c.680A>G(N227S), c.681T>G(N227K), c.689T>G(V230G), c.1604G>A(R535H)
12/F	GD1	c.655A>G(T219A)/RecNcil
13/F	GD1	c.226T>G(F76V)/c.596T>A(p.(L199Q)
14/M	GD1	c.226T>G(F76V)/c.1066delC
15/F	GD1	c.259C>T(R87W)/RecNcil
16/F	GD1	c.259C>T(R87W)/c.1448T>C(L483P)
17/F	GD1	c.226T>G(F76V)/c.1448T>C(L483P)
18/M	GD1	c.1448T>C(L483P)/c.1448T>C(L483P)
19/M	GD1	c.254G>A(G85E)/RecNcil
20/F	GD1	c.475C>T(R159W)/c.1240G>C(V414L)
21/F	GD1	c.1448T>C(L483P)/c.1504C>T(R502C)
22/M	GD1	c.721G>A(G241R)/c.1504C>T(R502C)
23/M	GD1	c.1066 delC(L356Wfs*8)/c.1174C>T(R392W)
24/M	GD1	c.260G>T (R87L)/c.1448T>C(L483P)
25/M	GD3	c.1342G>C(D448H)/c.1448T>C(L483P)
26/F	GD3	c.1448T>C(L483P)/c.1448T>C(L483P)
27/F	GD1	c.680A>G(N227S), c.681T>G(N227K), c.689T>G(V230G)/c.907C>A(L303I)
28/M	GD2-3	c.604C>T(R202X)/c.1603C>T(R535C)
29/F	GD1	c.890A>G(D297G)/c.680A>G(N227S), c.681T>G(N227K), c.689T>G(V230G), c.703T>C(S235P), c.721G>A(G241R)
30/F	GD1	c.1448T>C(L483P)/c.1448T>C(L483P)
31/F	GD1	c.1603C>T(R535C)/c.1448T>C(L483P)
32/F	GD1	c.1448T>C(L483P)/c.1448T>C(L483P)

Abbreviations: F, female; GBA, glucocerebrosidase; GD 1, type 1 Gaucher disease; GD 2-3, Gaucher disease intermediate type 2-3; GD 3, type 3 Gaucher disease; RecNcil, [c.1448T>C (p.L483P), c.1483G>C (p.A495P), c.1497G>C (p.V499 V)]; M, male.

To further analyze the response of ambroxol among patients, the baseline and final hemoglobin, platelet, and biomarker levels were analyzed in subgroups according to the age at starting treatment: younger than 18 years (mean [SD] age, 6.3 [2.7] years; n = 15) and 18 years or older (mean [SD] age, 31.3 [8.3] years; n = 6).

### Hematologic and Visceral Outcomes

**Figure 1.**
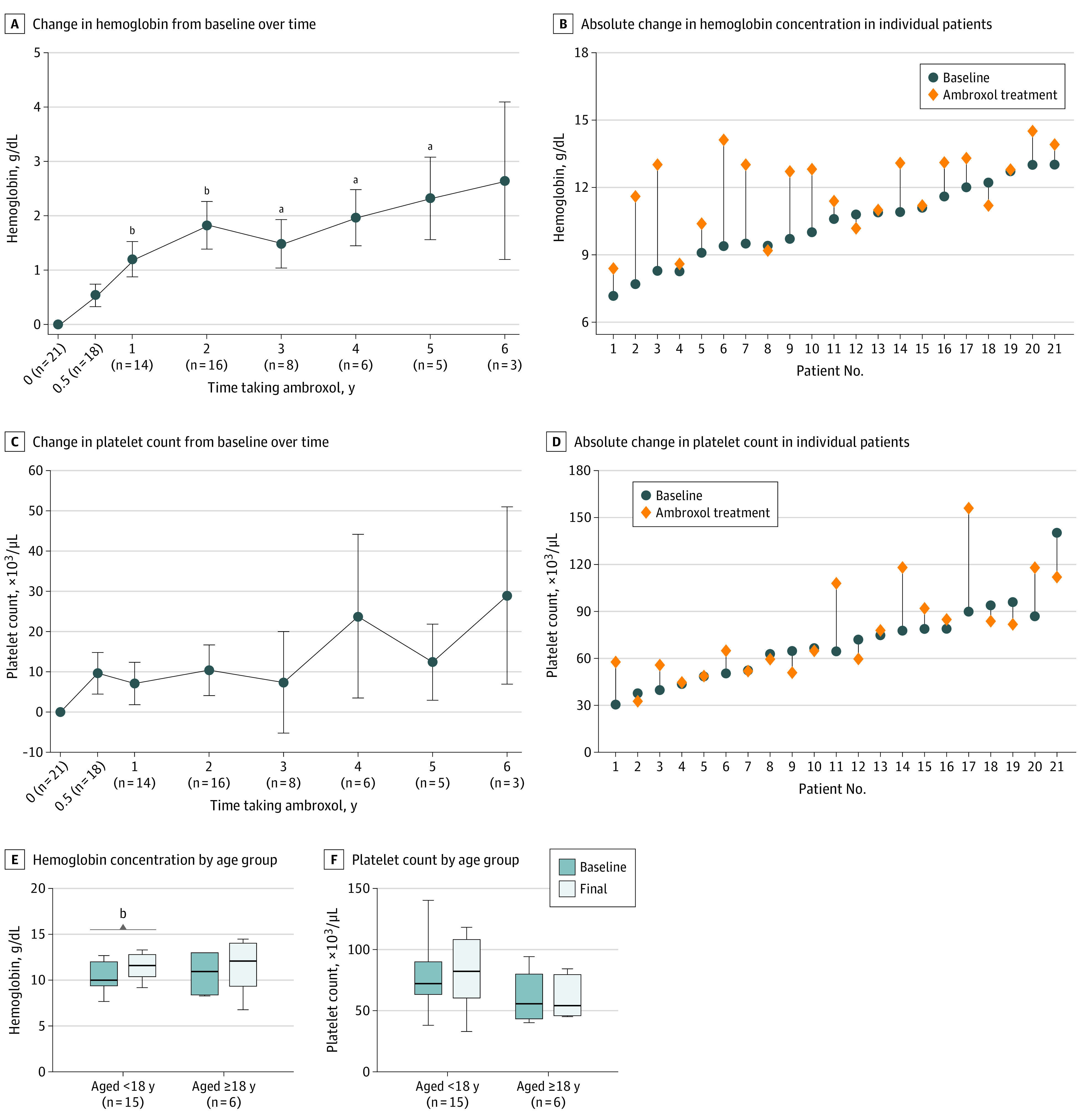
Hematologic Improvement During Ambroxol Therapy Hematologic improvement during ambroxol therapy excludes 5 splenectomized patients. A and C, Error bars indicate SE of the mean. No comparative analysis was performed at the time point with a 6-year treatment duration due to the small number of patients (n = 3). E and F, Whiskers indicate the minimum and maximum range; the line inside the box indicates the median value. To convert hemoglobin to grams per liter, multiply by 10.0; and platelets to ×10^9^ per liter, multiply by 1.0. ^a^*P* < .05. ^b^*P* < .01.

An increase in hemoglobin level was observed 1 year after the initiation of ambroxol. After a mean (SD) of 2.6 (1.7) years of ambroxol, the mean (SD) hemoglobin concentration improved by 14.4% (from 10.4 [1.7] to 11.9 [1.7] g/dL; mean [SD] change, 1.6 [1.7] g/dL; 95% CI, 0.8-2.3 g/dL; 2-sided paired *t* test, *P* < .001). A total of 18 of 21 patients (85.7%) had improved hemoglobin concentrations at the last clinic visits (Figure 1A and B). Wide variation was noted in the change in platelet count; the mean (SD) platelet count ranged from 69 (25) to 78 (30) × 10^3^/µL (mean [SD], 9 [22] × 10^3^/µL; 95% CI, −2 to 19 × 10^3^/µL; 2-sided paired *t* test; *P* = .09) (Figure 1C and D). A total of 16 of 21 patients (76.2%) met the hemoglobin goal, and 5 of 21 (23.8%) met the platelet goal.

The mean (SD) white blood cell count ranged from 4800 (2040) to 5150 (1890) cells/μL (mean [SD], 350 [1650] cells/uL; 95% CI, −400 to 1100 cells/μL [to convert to ×10^9^ per liter, multiply by 0.001]; 2-sided paired *t* test, *P* = .34; eFigure 2 in Supplement 1). Seven patients had low white blood cell count at baseline; the mean (SD) white blood cell count for these 7 patients increased by 45.2% (from 2390 [650] to 3470 [1360] cells/uL; mean [SD] change, 1080 [940] cells/uL; 2-sided paired *t* test, *P* = .02) at the last visit.

In subgroup analysis according to age at starting treatment, the mean (SD) hemoglobin concentration for patients who began treatment when they were younger than 18 years increased by 16.5% (from 10.3 [1.5] to 12.0 [1.5] g/dL; mean [SD] change, 1.6 [1.6] g/dL; 95% CI, 0.7-2.5 g/dL; 2-sided paired *t* test, *P* = .002), while the mean (SD) hemoglobin concentration increased by 12.3% (from 10.6 [2.4] to 11.9 [2.3] g/dL; mean [SD] change, 1.3 [1.8] g/dL; 95% CI, −0.6 to 3.2 g/dL; 2-sided paired *t* test, *P* = .14) for patients who began treatment when they were 18 years or older. For patients who began treatment when they were younger than 18 years, the mean (SD) platelet count increased by 12.0% (from 75 [24] to 84 [33] × 10^3^/µL; mean [SD] change, 9 [26] × 10^3^/µL; 95% CI, −5 to 24 × 10^3^/µL; 2-sided paired *t* test, *P* = .17), and for patients who began treatment when they were 18 years or older, the mean (SD) platelet count increased by 10.7% (from 56 [24] to 62 [15] × 10^3^/µL; mean [SD] change, 6 [13] × 10^3^/µL; 95% CI, −8 to 20 × 10^3^/µL; 2-sided paired *t* test, *P* = .32) (Figure 1E and F).

**Figure 2.**
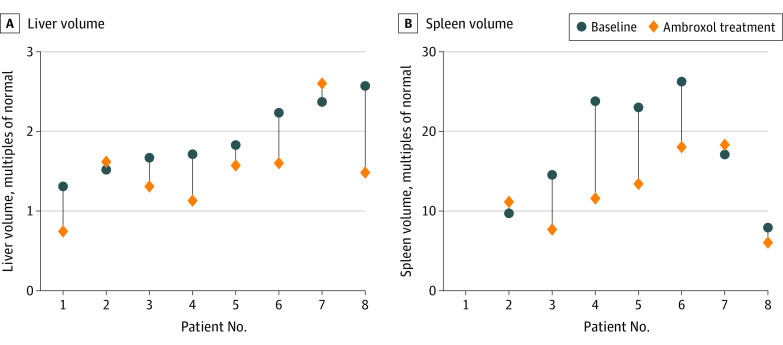
Absolute Spleen and Liver Volume Changes in Individual Patients Liver and spleen volume data before and after treatment were available for only 8 patients. Patient 1 was splenectomized. Due to the expense and time spent on testing, some patients did not undergo abdominal imaging to monitor spleen and liver volumes throughout therapy.

Spleen and liver volume changes for individual patients are shown in Figure 2. The mean (SD) spleen volume decreased by 29.5% (from 17.47 [7.18] to 12.31 [4.71] MN; mean [SD] change, −5.16 [5.44] MN; 95% CI, −10.19 to −0.13 MN; 2-sided paired *t* test, *P* = .04), and the mean (SD) liver volume decreased by 21.1% (from 1.90 [0.44] to 1.50 [0.53] MN; mean [SD] change, −0.39 [0.42] MN; 95% CI, −0.75 to −0.04 MN; 2-sided paired *t* test, *P* = .03). Three of 7 patients (42.9%) met the spleen volume goal, and 6 of 8 patients (75.0%) met the liver volume goal.

### Biomarkers in Plasma Before and During Ambroxol Treatment

**Figure 3.**
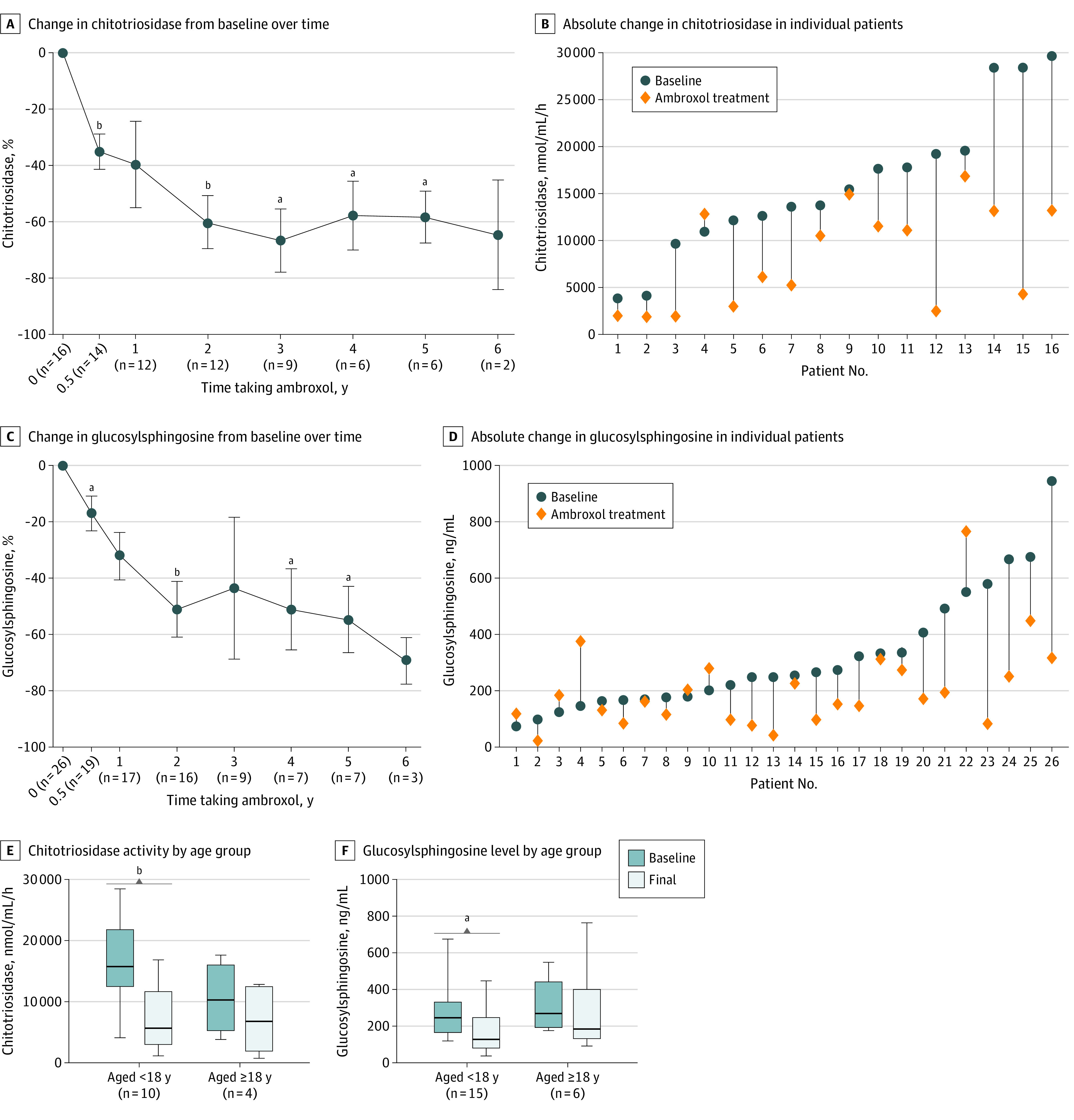
Changes From Baseline in Disease-Related Biomarkers During Ambroxol Treatment Chitotriosidase analysis excluded 10 patients with no chitotriosidase activity. A and C, Error bars indicate SE of the mean. No comparative analysis was performed at the time point with a 6-year treatment duration due to the small number of patients (n ≤ 3). E and F, 5 splenectomized patients were excluded; whiskers indicate the minimum and maximum range; the line inside the box indicates the median value. ^a^*P* < .05. ^b^*P* < .01.

Reductions in glucosylsphingosine level and chitotriosidase activity occured after the initiation of ambroxol, and the reductions were maintained (Figure 3A and C). Chitotriosidase activity was decreased for 15 of 16 patients (93.8%), and 20 of 26 patients (76.9%) had reduced glucosylsphingosine levels. The median chitotriosidase activity decreased by 43.1% (from 14 598 [range, 3849-29 628] to 8312 [range, 1831-16 842] nmol/mL/h; *z* = −3.413; 2-sided Wilcoxon test, *P* = .001), and the median glucosylsphingosine level decreased by 34.1% (from 251.3 [range, 73.6-944.2] to 165.7 [range, 21.3-764.8] ng/mL; *z* = −2.756; 2-sided Wilcoxon test, *P* = .006) for a mean (SD) of 2.6 (1.7) years of ambroxol treatment (Figure 3B and D). Of 26 patients, 11 received relatively long-term ambroxol treatment (mean [SD], 4.5 [1.5] years); these patients had larger reductions from baseline in chitotriosidase activity (–77.8% [from 13 580 to 3028 nmol/mL/h]; *z* = −2.666; 2-sided Wilcoxon test, *P* = .008) and glucosylsphingosine level (–52.8% [from 276.1 to 130.2 ng/mL]; *z* = −2.934; 2-sided Wilcoxon test, *P* = .003).

Splenectomized patients had a robust treatment response. Of the 5 splenectomized patients (cases 3, 13, 18, 24, and 27), case 13 had a comparatively mildly elevated glucosylsphingosine level (73.7 ng/mL) at baseline and showed relatively stable glucosylsphingosine levels over the treatment period, with −42.7% liver volume. Biomarker improvement was noted among all other splenectomized patients, with decreased glucosylsphingosine levels ranging from −16.9% to −77.9% and decreased chitotriosidase activity ranging from −24.2% to −55.4%.

When biomarkers were analyzed in subgroups according to age at starting treatment, the median chitotriosidase activity among patients who began treatment when they were younger than 18 years decreased by 64.0% (from 15 710 [range, 4092-28 422] to 5658 [range, 1146-16 843] nmol/mL/h; *z* = −2.803; 2-sided Wilcoxon test, *P* = .005) and by 34.6% (from 10 276 [range, 3849-17 607] to 6724 [range, 1905-12 790] nmol/mL/h; *z* = −1.461; 2-sided Wilcoxon test, *P* = .14) among patients starting treatment when they were 18 years or older (Figure 3E). The median glucosylsphingosine level for patients beginning treatment when they were younger than 18 years decreased by 47.3% (from 248.5 [range, 122.8-674.9] to 131.0 [range, 41.1-448.5] ng/mL; *z* = −2.385; 2-sided Wilcoxon test, *P* = .02) and by 30.9% (from 271.3 [range, 179.8-549.7] to 187.4 [range, 95.8-764.8] ng/mL; *z* = −0.524; 2-sided Wilcoxon test, *P* = .60) for patients starting treatment when they were 18 years or older (Figure 3F). Larger decreases in chitotriosidase activity and glucosylsphingosine level were observed among patients who received treatment at a younger age.

## Discussion

Previous studies reported that ambroxol had a promising outcome when used with ERT or SRT for patients with GD.^11,16,17,19^ However, to our knowledge, there have been few reports on the clinical benefits of ambroxol alone for patients with GD.^14,20^ Our laboratory previously reported the early beneficial outcomes of ambroxol on skeletal and hematologic manifestations in a child with GD1.^20^ Here, we report observational data on ambroxol repurposing among patients without GD-specific treatments.

Some *GBA1* pathogenic variants are associated with relatively mild symptoms. The R392W, F76V, N227S, R87W, M75V, R159W, L188F, L303I, V414L, and R502C variants had previously been associated with a late onset and mild phenotype; therefore, these variants were classified as mild.^21,22,23,24^ Most of the 26 individuals in the study who were considered good responders to ambroxol carried 1 or 2 mild variants. Previous research^21,22,25^ showed variants L483P, RecNcil, R535C, and R202X, and gene conversion with *GBAP* (OMIM 606463) N227S, N227K, and V230G were associated with nGD or severe GD1 phenotype. Some patients with 1 severe variant and 1 moderate variant, such as L483P heterozygote with another mild variant (cases 2-4 and case 21), as well as 1 patient with RecNcil/G85E (case 19), responded favorably to ambroxol. We hypothesized that there could be benefits associated with ambroxol among patients with *GBA1* variants associated with some degree of residual β-glucocerebrosidase activity.

Case 28 had a severe R535C-R202X genotype and was classified as having GD intermediate type 2-3. Case 11 had genotype gene conversion with *GBAP* N227S, N227K, V230G, R535H, and R87Q and received a diagnosis of severe GD1 phenotype. These 2 patients were at significantly advanced stages of GD at baseline (eg, case 11 had a spleen volume of 41 MN; and poor hematologic parameters; case 28 had a spleen volume of 87 MN; and horizontal gaze palsy), with deterioration of hematologic parameters and biomarkers, showed no improvement in clinical presentation, and ambroxol treatment was considered nonefficacious. We believe that in the clinical course of GD, there is a critical threshold beyond which ambroxol therapy may not be beneficial as previously described for nGD.^26^ Thus, early initiation of ambroxol therapy will be critical for the progression of the disease. Another therapeutic modality may act synergistically to better control disease progression.

Results of a study involving 1016 patients with GD1 who received imiglucerase therapy for 4 to 5 years suggested that platelets increased only when the spleen volume decreased substantially.^27^ In our study, platelets responded to ambroxol less favorably and showed a smaller increase than hemoglobin. The relatively poor platelet responses might be associated with the large spleen volumes. As shown in Figure 2, for almost all patients, despite substantial improvement from baseline, spleen volumes continued to exceed 10 MN.

Due to the expense and time spent on testing, some patients did not undergo abdominal imaging to monitor spleen and liver volumes. In a large cohort of patients with type 1 GD treated with imiglucerase for 2 years, mean spleen and liver volumes were reduced by 49% and 29% from baseline, respectively, according to retrospective research from the International Collaborative Gaucher Group Gaucher Registry.^28^ Although ambroxol therapy did not improve visceral outcomes as significantly as ERT, it was still encouraging.

Glucosylsphingosine, a downstream metabolic product of glucosylceramide, is increasingly recognized as a sensitive biomarker of GD with direct involvement in disease pathogenesis.^29^ Monitoring chitotriosidase activity in patients with GD1 has limitations since it is subject to genetic heterogeneity.^30^ Nearly 40% of patients in this study had no chitotriosidase activity. Glucosylsphingosine level could be used to assess the efficiency of ambroxol in patients with no chitotriosidase activity. A significant decrease in glucosylsphingosine level was observed in the patients. However, it was reported that glucosylsphingosine level decreased more substantially in patients treated with ERT or SRT.^31,32^ The median glucosylsphingosine level (327.0 ng/mL [range, 123.6 to 482.6 ng/mL]) was reduced by 85.7% in patients with GD1 who completed 209 weeks of velaglucerase alfa treatment.^31^ In a study of patients with GD1 receiving eliglustat therapy for 8 years, glucosylsphingosine was decreased approximately 60% from baseline at 1 year and 92% from baseline at 8 years.^32^ The degree of decrease in glucosylsphingosine level might be associated with the overall disease severity of the patients. Several reports demonstrated an additional decrease in glucosylsphingosine level after ambroxol therapy was started for patients treated with long-term ERT.^16,33^

Patients with younger ages at the start of treatment had larger improvements in hematologic parameters and plasma biomarkers. Early administration of ambroxol might achieve better outcomes for patients with early-stage GD. Consequently, earlier intervention is advisable for patients with GD.

In rodent and primate models, ambroxol increases intracellular β-glucocerebrosidase activity in tissues, including the brain.^34,35^ In our study, neurologic stability or improvement was observed in 2 patients with GD type 3 (cases 25 and 26; eTable in Supplement 1), which suggests that the therapeutic outcomes of ambroxol might delay the rapid natural progression of neurologic manifestations. However, due to sampling difficulty, we did not have data on changes in glucosylsphingosine level in the cerebrospinal fluid, which has previously been reported to be decreased considerably in patients with GD3 after ambroxol treatment.^11,15^ A recent nonrandomized, noncontrolled trial of ambroxol for patients with Parkinson disease demonstrated that ambroxol could cross the BBB and have a modulatory association with the β-glucocerebrosidase enzyme.^15^ Ambroxol increases β-glucocerebrosidase enzyme protein levels and cerebrospinal fluid α-synuclein levels, thus explaining at least part of the clinical improvement observed in patients with nGD.

Two patients in our study (case 3 and case 20) who experienced a stable clinical course without deterioration had no significant improvement in biomarkers during early ambroxol therapy. After continuous ambroxol treatment for 4 years, a marked reduction in glucosylsphingosine level and chitotriosidase activity was observed. This evidence indicates that ambroxol may take longer to reverse the pathologic glycosphingolipid accumulation in GD. Therefore, it is important that patients take their medication consistently.

Most patients in this cohort were from provinces other than Shanghai. It is difficult for some patients to visit our hospital regularly due to time and cost reasons, particularly for patients living in remote rural areas. Clinical improvements were observed in 4 patients (cases 5, 6, 16, and 17) with mild gene variants, but biomarkers were volatile or did not improve significantly at follow-up visits. These findings might be associated with the patients’ irregular use of ambroxol recorded in the medical history as reported by parents at follow-up.

In this study, patients received a high dose of oral ambroxol for 0.5 to 6.5 years without significant adverse effects, and most of the adverse events were mild and transient. Hence, these findings also provide additional evidence of the long-term safety of ambroxol.

### Limitations

Our study has several limitations. Almost all the patients involved in this study were from cities other than Shanghai or from distant rural areas. It was difficult for some patients to arrive at the center on time due to the impact of COVID-19 over the past 3 years. In this case, the number of patients at different follow-up time points were incomplete. Patient adherence is an important factor for a drug that must be taken regularly to exert its effect. Some studies tested serum and cerebrospinal fluid ambroxol concentrations to assess adherence to treatment.^11,15^ The serum concentration of ambroxol should be measured to assess participant adherence to treatment. It might be helpful not only for analysis of clinical outcomes but also for drug dose adjustment for patients with GD.

## Conclusions

In this case series of ambroxol repurposing for patients with GD, long-term treatment with ambroxol was safe and associated with patient improvement. This improvement was larger in patients with mild symptoms and patients who began treatment at younger ages. Ambroxol can be an option when ERT or SRT is not available or affordable. We expect that these data may encourage physicians and patients to consider the off-label use of ambroxol for GD.

## Notes

Supplement 1. **eFigure 1.** Flow Diagram of Participant Recruitment and Retention **eTable.** Detailed Data of the 32 Patients Included in the Study **eFigure 2.** White Blood Cell Count During Ambroxol Therapy

Supplement 2.
Data Sharing Statement
