# Supplementary material for: Use of Ambroxol as Therapy for Gaucher Disease
Source: JAMA Netw Open. 2023 Jun 21;6(6):e2319364. doi: 10.1001/jamanetworkopen.2023.19364 (PMC10285580; doi:10.1001/jamanetworkopen.2023.19364)
Supplement: Supplement 1. — eFigure 1. Flow Diagram of Participant Recruitment and Retention eTable. Detailed Data of the 32 Patients Included in the Study eFigure 2. White Blood Cell Count During Ambroxol Therapy [file jamanetwopen-e2319364-s001.pdf]

Supplementary Online Content

Zhan X, Zhang H, Maegawa GHB, et al. Use of ambroxol as therapy for Gaucher disease. *JAMA Netw Open.* 2023;6(6):e2319364. doi:10.1001/jamanetworkopen.2023.19364

eFigure 1. Flow Diagram of Participant Recruitment and Retention
eTable. Detailed Data of the 32 Patients Included in the Study
eFigure 2. White Blood Cell Count During Ambroxol Therapy

This supplementary material has been provided by the authors to give readers additional information about their work.

© 2023 Zhan X et al. *JAMA Network Open.*

eFigure 1. Flow Diagram of Participant Recruitment and Retention

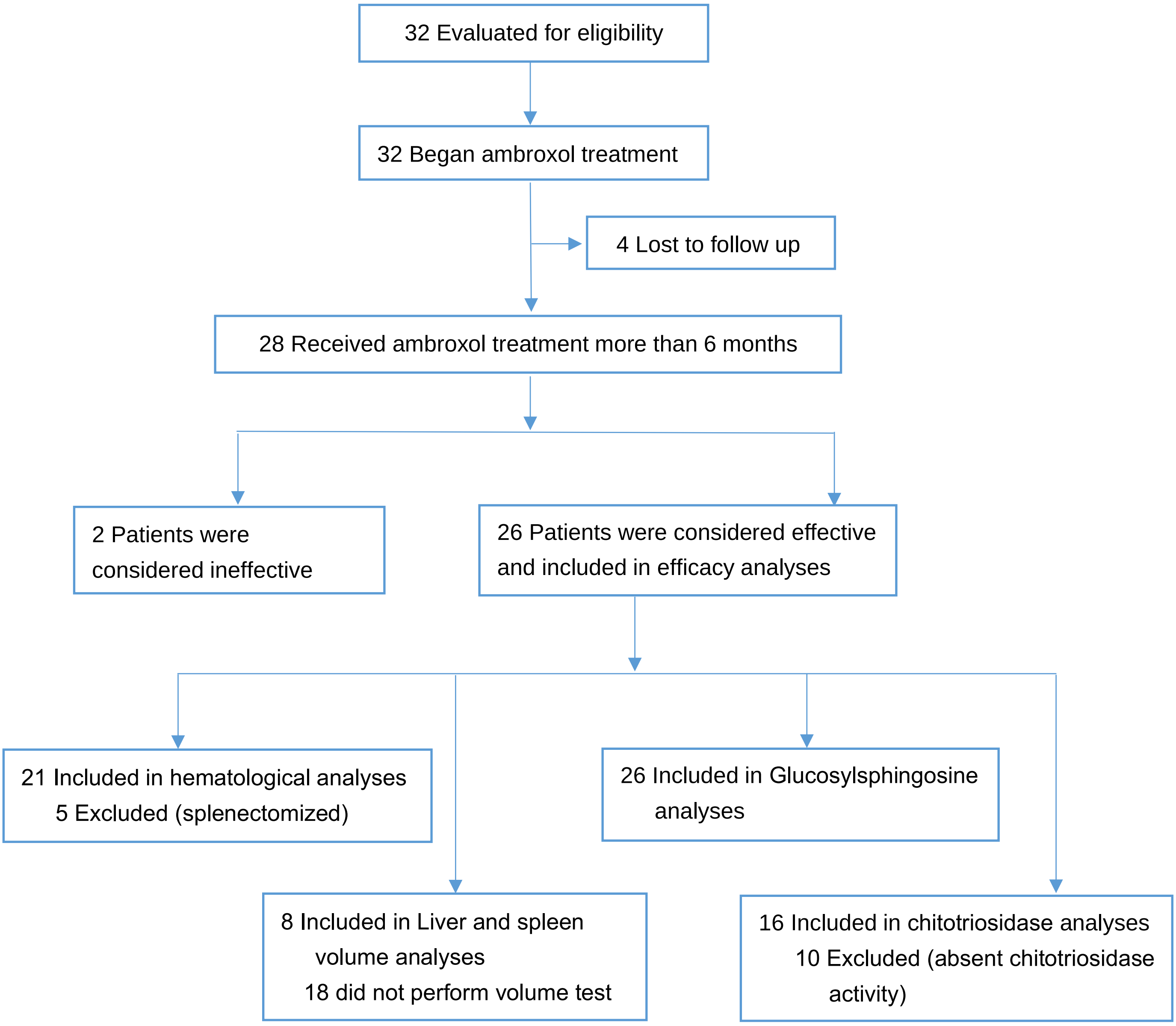

Twenty-one patients were included in hematological analysis, excluding five splenectomized patients. Sixteen patients were included in chitotriosidase analysis, excluding 10 patients with absent chitotriosidase activity. Liver and spleen volume analysis of pre- and post-treatment was available for 8 patients.

© 2023 Zhan X et al. *JAMA Network Open.*

eTable. Detailed Data of the 32 Patients Included in the Study

Case	Sex	Type	Clinical feature	Age at start of ambroxol (years)	Duration months	Dose (mg/Kg.day)	Adverse effects	Evidence of efficacy
1	F	GD1	Severe HS, A, T, B	6	70	16.8	-	Decrease of plasma lyso-Gb1 and CHIT activity, liver and spleen volume decrease, excellent academic performance.
2	M	GD1	Severe HS, severe T	29	60	14.5	-	Decrease of plasma plasma lyso-Gb1 and CHIT activity, hematological improvement.
3	M	GD1	Hepatomegaly, splenectomized	46	54	15.2	-	Decrease of plasma lyso-Gb1 and CHIT activity
4	M	GD1	Severe HS, severe T	47	52	6.4	-	Decrease of plasma lyso-Gb1 and CHIT activity, respond well and continuously.
5	M	GD1	Severe HS, A, B	2.5	39	13.5	-	Decrease of plasma CHIT activity, increased height, reduced severity and frequency of bone pain, no progressive enlargement of the liver and spleen.
6	F	GD1	Severe HS, severe A, T	23	74	13.7	-	Hematological improvement, decrease of plasma lyso-Gb1, increased physical activity without fatigue, no bleeding when brushing teeth, normal menstruation.
7	M	GD1	Severe HS, T	10	8	18.8	-	Decrease of plasma lyso-Gb1 and CHIT activity, reduced frequency of nose bleeding.
8	M	GD1	Severe HS, A, T	5	66	9	-	Decrease of plasma lyso-Gb1, CHIT activity, and liver and spleen volume, hematological improvement, and reduced frequency of bone pain and nose bleeding.

© 2023 Zhan X et al. *JAMA Network Open.*

Case	Sex	Type	Clinical feature	Age at start of ambroxol (years)	Duration months	Dose (mg/Kg.day)	Adverse effects	Evidence of efficacy
9	M	GD1	Severe HS, A, severe T	11	10	9.6	-	Decrease of plasma lyso-Gb1, hematological improvement, normal physical activity without fatigue, no bone pain.
10	F	GD1	Severe HS, B, severe A, severe T	32	24	8.7	-	hematological improvement, normal menstruation.
11	F	GD1	Severe HS, severe A, severe T	2	19	20	-	No effect [a]
12	F	GD1	Severe HS, severe A, T	7	6	10	-	Hematological improvement, decrease of liver and spleen volume.
13	F	GD1	Hepatomegaly, splenectomized	30	24	12.8	-	Decrease of liver volume, improved menstruation.
14	M	GD1	HS, A, T	5	50	20	-	Decrease of plasma lyso-Gb1 and CHIT activity, liver volume and spleen volume decrease, hematological improvement.
15	F	GD1	Severe HS, A, T	4.5	12	16.9	-	Decrease of plasma lyso-Gb1 and CHIT activity, hemoglobin increase.
16	F	GD1	Severe HS, T	27	12	5.4	Nausea	petechia disappear.
17	F	GD1	Severe HS, A, Severe T	30	24	6.5	-	Decrease of plasma CHIT activity, petechia and swelling of both lower limbs subsided .

© 2023 Zhan X et al. *JAMA Network Open.*

Case	Sex	Type	Clinical feature	Age at start of ambroxol (years)	Duration months	Dose (mg/Kg.day)	Adverse effects	Evidence of efficacy
18	M	GD1	Hepatomegaly, B, T, splenectomized	33	19	12	-	Decrease of DBS lyso-Gb1 and CHIT activity, hematological improvement, increased physical capacity.
19	M	GD1	Severe HS, severe A, T	6	27	15	Diarrhea, skin rash	Decrease of plasma lyso-Gb1 and CHIT activity, and liver and spleen volume,hematological improvement.
20	F	GD1	Severe HS, Severe T	2	50	13.5	-	Decrease of plasma lyso-Gb1 and CHIT activity, and liver and spleen volume, hematological improvement.
21	F	GD1	Hepatomegaly, Severe splenomegaly, A, T, B	7	39	10.7	-	Decrease in plasma lyso-Gb1 levels and CHIT activity, liver and spleen volume decrease, hematological improvement, reduced frequency of bone pain, catch-up growth.
22	M	GD1	Severe HS, A, T	4	8	11.1	-	Reduced frequency of nose bleeding, decrease of plasma CHIT activity.
23	M	GD1	HS, T	8	8	9.2	-	Decrease in plasma lyso-Gb1.
24	M	GD1	B, splenectomized	24	19	5.8	-	Decrease in plasma lyso-Gb1.
25	M	GD3	Hepatomegaly, Severe splenomegaly, severe T, E, O	11	32	15.5	-	Decrease in plasma lyso-Gb1, hematological improvement, neurological improvement [b].

© 2023 Zhan X et al. *JAMA Network Open.*

Case	Sex	Type	Clinical feature	Age at start of ambroxol (years)	Duration months	Dose (mg/Kg.day)	Adverse effects	Evidence of efficacy
26	F	GD3	Severe HS, A, severe T, E, O	7	28	13.5	-	Decrease of plasma lyso-Gb1, hematological improvement, reduced frequency of nose bleeding, and increased physical activity capability.
27	F	GD1	B, splenectomized	53	23	11.7	-	Decrease plasma in lyso-Gb1.
28	M	GD 2-3	Severe HS, T, O, strabismus, growth retardation	3	30	15.3	salivation	No effect [c]
29	F	GD1	Severe splenomegaly, severe T, lost to follow-up	57	Not available	10	Not available	Not available
30	F	GD1	HS, severe A, lost to follow-up	2	Not available	15	Not available	Not available
31	F	GD1	HS, A, T, lost to follow-up	2	Not available	15	Not available	Not available
32	F	GD1	Severe HS, A, severe T	13	Not available	14.4	Not available	Not available

F, female; M, Male; GD 1, type 1 Gaucher Disease; GD 3, type 3 Gaucher Disease; GD 2-3, intermediate type 2-3; HS, hepatosplenomegaly; A, amemia; T, thrombocytopenia; B, bone involvement; E, abnormal EEG; O: ophthalmoplegia; CHIT: chitotriosidase; lyso-Gb1: glucosylsphingosine; DBS: dried blood spot; severe thrombocytopenia: <60 *10^9/L; severe amemia: <9 g/dL.

© 2023 Zhan X et al. *JAMA Network Open.*

- : No adverse effects were described by the patients.

Expanded information on efficacy by patient number:

[a] Case 11: Massive splenomegaly (spleen volume 41 MN at baseline), poor hematologic parameters, increased lyso-Gb1 levels, enlarged abdomen.

[b] Case 25: Improved horizontal eye movement, improved head control when looking left and right.

[c] Case 28: Massive splenomegaly (spleen volume 87 MN at baseline), poor hematologic parameters, increased lyso-Gb1 levels, horizontal gaze palsy.

The height grew slowly in the past year. No improvement in speech and gait.

© 2023 Zhan X et al. *JAMA Network Open.*

eFigure 2. White Blood Cell Count During Ambroxol Therapy

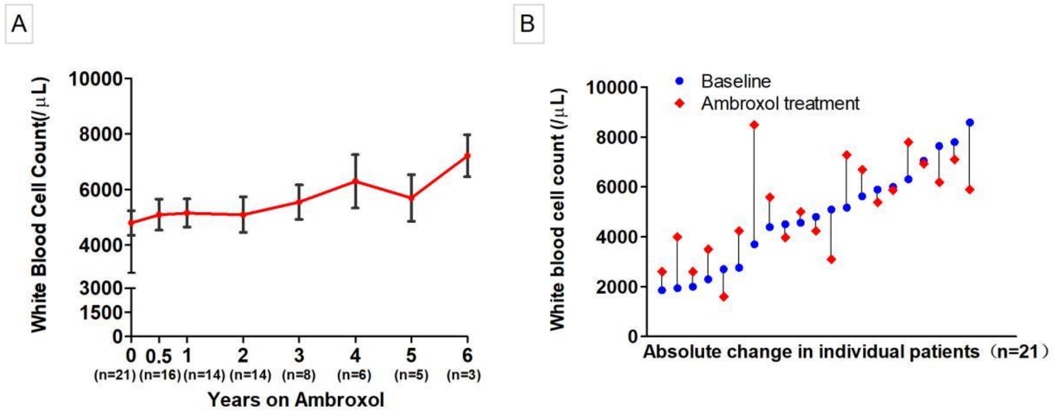

Analysis of white blood cell excluded 5 splenectomized patients, white blood cell count was listed as /μL, to convert to $\times 10^9$/L, multiply by 0.001. (A) White blood cell count during ambroxol therapy. Error bars indicate SE of the mean. (B) Absolute White blood cell count change in individual patients.

© 2023 Zhan X et al. *JAMA Network Open.*
