# Supplementary material for: Use of Ambroxol as Therapy for Gaucher Disease
Source: JAMA Netw Open. 2023 Jun 21;6(6):e2319364. doi: 10.1001/jamanetworkopen.2023.19364 (PMC10285580; doi:10.1001/jamanetworkopen.2023.19364)
Supplement: Supplement 2. — Data Sharing Statement [file jamanetwopen-e2319364-s002.pdf]

Data Sharing Statement

Zhan. Use of Ambroxol as Therapy for Gaucher Disease. *JAMA Netw Open*. Published June 21, 2023. doi:10.1001/jamanetworkopen.2023.19364

Data
Data available: No

Additional Information
Explanation for why data not available: The datasets generated and/or analysed during the current study are available from the corresponding author on reasonable request
